# Development of a Gut-on-a-Chip Model for High Throughput Disease Modeling and Drug Discovery

**DOI:** 10.3390/ijms20225661

**Published:** 2019-11-12

**Authors:** Claudia Beaurivage, Elena Naumovska, Yee Xiang Chang, Edo D. Elstak, Arnaud Nicolas, Heidi Wouters, Guido van Moolenbroek, Henriëtte L. Lanz, Sebastiaan J. Trietsch, Jos Joore, Paul Vulto, Richard A.J. Janssen, Kai S. Erdmann, Jan Stallen, Dorota Kurek

**Affiliations:** 1 Galapagos BV, Zernikedreef 16, 2333 CL Leiden, The Netherlands; claudia.beaurivage@glpg.com (C.B.); y.x.chang@students.uu.nl (Y.X.C.); edo.elstak@glpg.com (E.D.E.); Heidi.Wouters-EXT@glpg.com (H.W.);; 2 Department of Biomedical Sciences, University of Sheffield, Western Bank, Sheffield S10 2TN, UK; e.naumovska@mimetas.com (E.N.); k.erdmann@sheffield.ac.uk (K.S.E.); 3 Mimetas BV, J.H. Oortweg 16, 2333 CH Leiden, The Netherlands; a.nicolas@mimetas.com (A.N.); g.t.van.moolenbroek@umail.leidenuniv.nl (G.v.M.); h.lanz@mimetas.com (H.L.L.); s.trietsch@mimetas.com (S.J.T.); j.joore@mimetas.com (J.J.); p.vulto@mimetas.com (P.V.)

**Keywords:** inflammation, inflammatory bowel disease, gut-on-a-chip, Organ-on-a-Chip, microfluidic, drug discovery, disease modeling

## Abstract

A common bottleneck in any drug development process is finding sufficiently accurate models that capture key aspects of disease development and progression. Conventional drug screening models often rely on simple 2D culture systems that fail to recapitulate the complexity of the organ situation. In this study, we show the application of a robust high throughput 3D gut-on-a-chip model for investigating hallmarks of inflammatory bowel disease (IBD). Using the OrganoPlate platform, we subjected enterocyte-like cells to an immune-relevant inflammatory trigger in order to recapitulate key events of IBD and to further investigate the suitability of this model for compound discovery and target validation activities. The induction of inflammatory conditions caused a loss of barrier function of the intestinal epithelium and its activation by increased cytokine production, two events observed in IBD physiopathology. More importantly, anti-inflammatory compound exposure prevented the loss of barrier function and the increased cytokine release. Furthermore, knockdown of key inflammatory regulators *RELA* and *MYD88* through on-chip adenoviral shRNA transduction alleviated IBD phenotype by decreasing cytokine production. In summary, we demonstrate the routine use of a gut-on-a-chip platform for disease-specific aspects modeling. The approach can be used for larger scale disease modeling, target validation and drug discovery purposes.

## 1. Introduction

Inflammatory bowel disease (IBD), including ulcerative colitis (UC) and Crohn’s disease (CD), is a complex chronic idiopathic disease severely incapacitating the life of more than 2.5 to 3 million Europeans, bringing a high socio-economic burden to society [1,2,3]. The aetiology of the disease remains elusive; however, it is known to involve the interaction of genetic, environmental, microbiological and immunological factors [3,4]. There is a general consensus in the scientific community that IBD arises from a dysregulated activation of immune effectors in response to commensal microbiota, which is triggered by environmental factors in a genetically-susceptible host [5,6]. Important and frequent primary events in IBD include loss of function of the epithelial barrier and an impaired balance between pro- and anti-inflammatory mediators secreted by intestinal epithelial cells (IECs) or immune cells [6,7].

The multifactorial nature of IBD makes it extremely difficult to develop realistic disease models that will be able to grasp and recapitulate the complexity of the disease development and progression. This is particularly important knowing that 25% to 30% of patients fail to respond to standard IBD therapy and more than 20% have to discontinue ongoing treatments due to unforeseen side-effects [8]. The need for deeper understanding of the disease’s mechanisms and for the development of new medicines is therefore urgent.

Different in vivo, ex vivo and in vitro models are currently used to study IBD aetiology [9,10,11,12]. Despite the interspecies difference, animal models have proven to be extremely useful to understand IBD mechanisms such as the essential role of microbiota and T cells [13,14]. However, manipulation of individual IBD parameters, such as barrier function of the epithelium and cell-type specific activation, are difficult to achieve in these complex models, which proves to complicate matters and in turn leads to poor prognosis of drug candidates [15]. Furthermore, from a drug discovery perspective, high throughput screening of compounds cannot be achieved in such models. On the other hand, ex vivo models such as intestinal explants come from a limited source with an extremely limited lifespan, making them unsuitable for drug discovery purposes [10,16,17]. Standard in vitro setups usually include the static culture of epithelial cells on rigid membranes separating two different chambers, sometimes comprising an immune component [18]. Such conventional membrane insert-based systems, often called 2.5D or Transwell systems, are a useful tool with medium throughput but do not recapitulate 3D cell organization, which has proven to be important for increased physiological relevance [19]. Given the limitations of existing models, there is a need for a tool that will mimic key aspects of IBD in a robust manner that is compatible with high throughput screening equipment.

The recently emerged field of Organ-on-a-Chip technology offers a potential alternative to traditional 2D cell cultures and animal models. Recent studies have shown enormous progress in both gut-on-a-chip and IBD research [20,21]. In most cases, chips made out of a silicon rubber material are seeded with either cell lines, induced pluripotent stem cells (iPSCs) or Lgr5^+^-derived organoids [22,23]. These studies are extremely valuable to assess the impact of micro engineering techniques on the physiological relevance of cell culture models. Nevertheless, there are some shortcomings associated with such silicon rubber chips including the lack of scalability and the reduction of bioavailability of some drugs and small molecules due to unspecific adsorption [24,25].

To overcome these limitations and to allow the integration of Organ-on-a-Chip in the drug development process, we further adapted a recently published gut-on-a-chip system [26] to mimic inflammatory conditions. This model, based on the use of Caco-2 cells, has already been proven to be easy-to-use, robust and reproducible as well as relatively fast; Caco-2 cells form 3D leak-tight polarized tubules with accessible apical and basal sides after only 4 days of culture. The platform we used in this study, the OrganoPlate, offers a high throughput alternative to silicon rubber chips and allows compound screening due to its glass composition. 

To mimic inflammatory characteristics in this model, we applied an optimised immune-relevant cytokine trigger that mimics the effect of *E. coli*-activated dendritic cells (DCs) on the IECs [27,28,29]. We assessed the effect of this trigger on two main aspects of IBD—the integrity of the intestinal barrier as well as the cellular activation of IECs. To assess barrier integrity, we measured transepithelial electrical resistance (TEER) values of Caco-2 tubules in a high throughput manner and assessed the localisation of cell junction-associated protein E-CADHERIN. The production of epithelial-relevant inflammatory cytokines by Caco-2 cells was used as a readout for cellular activation following trigger. 

To assess the applicability of the model for target and drug discovery processes, we performed direct on-chip adenoviral transduction of validated shRNAs against proinflammatory targets RELA and MYD88. We further addressed the ability of the model to respond to a well-known anti-inflammatory compound, TPCA-1 [30,31]. Overall, our results show that this 3D model can be used as an in vitro tool for drug development in an accelerated manner and opens the way to more physiologically relevant models usable in high throughput experiments. 

## 2. Results

### 2.1. Establishing Leak-Tight 3D Caco-2 Tubules Controlled by TEER Measurements

**Figure 1:**
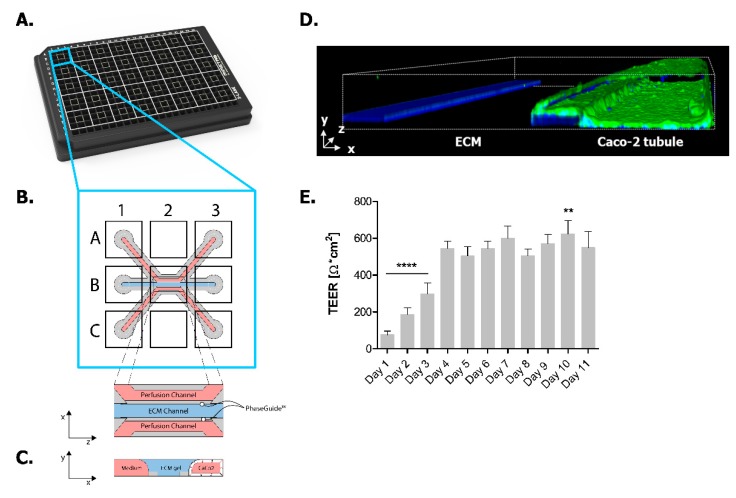
Caco-2 tubules in the 3-lane OrganoPlate. (**A**) Photograph of the 3-lane OrganoPlate. Each plate contains 40 individual microfluidic chips. (**B**) Schematic representation of a microfluidic chip; each chip has three microfluidic channels each containing two medium channels (pink) and a gel channel (blue). Each channel has an inlet (A1, B1 and C1) and an outlet (A3, B3 and C3). Real-time imaging is done through the observation window (B2). (**C**) Transversal view of a microfluidic view; Caco-2 cells adhere to the ECM meniscus created by the PhaseGuide™ technology. Upon medium perfusion, Caco-2 cells form tubules covering the walls of the top channel. (**D**) 3D reconstruction image of a Caco-2 tubule at Day 4 stained for ACTIN (green) and DNA (blue), depicting the nuclei. (**E**) Transepithelial electrical resistance (TEER) values of Caco-2 tubules over time until Day 11. Data is represented as mean ± SEM. ** *p* < 0.01; **** *p* < 0.0001 by one-way ANOVA with Dunnett’s post-hoc test compared to Day 4 (*n* = 13).

In this study, we employed the OrganoPlate platform in order to establish a 3D in vitro model recapitulating key IBD characteristics. We successfully established this IBD model by adapting our previously published gut-on-a-chip model where Caco-2 cells grow in 3D tubules following medium perfusion [26]. Figure 1 shows the 3-lane OrganoPlate where 40 microfluidic chips are organized at the bottom side of a standard 384-well plate format (Figure 1A). Each of these chips has three microfluidic channels with dedicated inlets and outlets as well as an observation window allowing real-time monitoring of the 3D culture (Figure 1B). An extracellular matrix (ECM) precursor is loaded into the middle channel and patterned with a surface tension technique called phase guiding [32]. After gelation, fluids can be inserted in adjacent channels, allowing membrane-free co-culture of several cell types. In our study, we seeded Caco-2 cells in the top channel against an ECM gel while the bottom channel was kept free of cells (Figure 1C). Data from our previous study showed that, upon medium perfusion, Caco-2 cells form a complete and polarized leak-tight tubule after 4 days of culture in OrganoPlate (Figure 1D). In those earlier studies, we determined the barrier integrity using a FITC-labeled dextran leakage assay [26]. In the current study, we assessed the barrier integrity by measuring the transepithelial electrical resistance (TEER) values of the Caco-2 tubules, leading to more sensitive and accurate data [33]. We show that the TEER values of Caco-2 tubules continuously increase and stabilise after 4 days of culture until the end of the experimentation (Figure 1E).

### 2.2. Induction of Inflammatory State in Caco-2 Tubules

**Figure A1:**
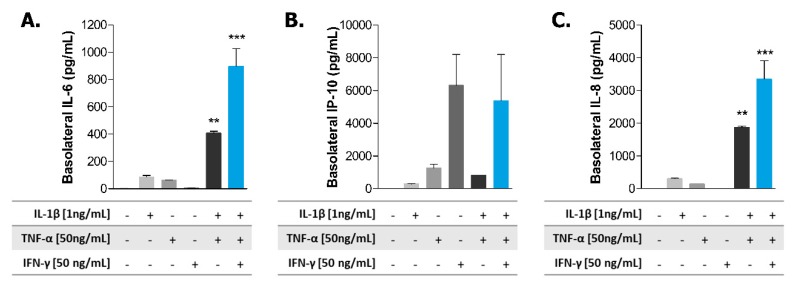
Optimization of the cytokine trigger composition in the Transwell system. Basal secretion of IL-6 (**A**), IP-10 (**B**) and IL-8 (**C**) by Caco-2 cells at Day 19 after a 72 h trigger with the indicated cytokines. Data is represented as mean ± SEM. * *p* < 0.05; ** *p* < 0.01; *** *p* < 0.001 by one-way ANOVA with Dunnett’s post-hoc test compared to non-triggered condition (*n* = 2).

**Figure A2:**
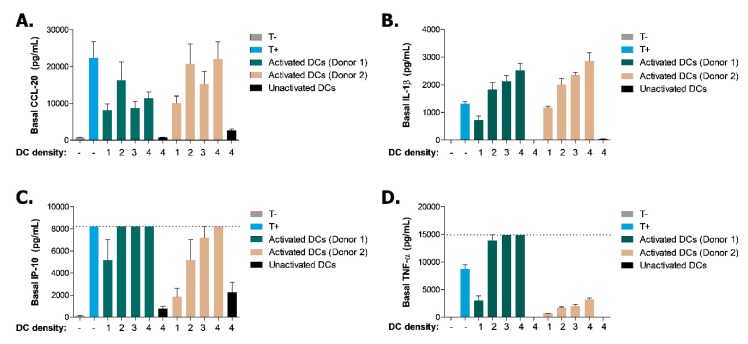
Effect of a cytokine trigger and the presence of activated-dendritic cells on the cytokine release of Caco-2 cells. Basal production of CCL-20 (**A**), IL-1β (**B**), IP-10 (**C**) and TNF-α (**D**) assessed by Luminex at Day 19. Caco-2 cells were either left non-triggered (T-) or triggered (T+) with cytokines IL-1ß, TNF-α and IFN-γ (1, 50, 50 ng/mL respectively) or co-cultured with *E. coli*-activated DCs from two donors (DC density; 1 = 20,000, 2 = 55,000, 3 = 90,000, 4 = 125,000) or non-activated DCs for 72 h. Data is represented as mean ± SEM. Dotted line depicts the maximum detection range of the assay (*n* = 2–4).

Based on previous literature [34], we optimised a cytokine cocktail that replicates the effect of *E. coli*-activated DCs on the cytokine secretion of Caco-2 cells in the Transwell system. We first optimised the composition of the trigger and found that a combination of IL-1β, TNF-α and IFN-γ led to the highest cytokine production in Caco-2 cells (Figure A1). We then compared the trigger’s effect to the effect of different concentrations of *E. coli*-activated DCs on the cytokine production of Caco-2 cells (Figure A2). The optimised trigger led to comparable cytokine production levels as the presence of *E. coli*-activated DCs by Caco-2 cells, supporting the immunological relevance of the cytokine cocktail as an inflammatory trigger.

**Figure 2:**
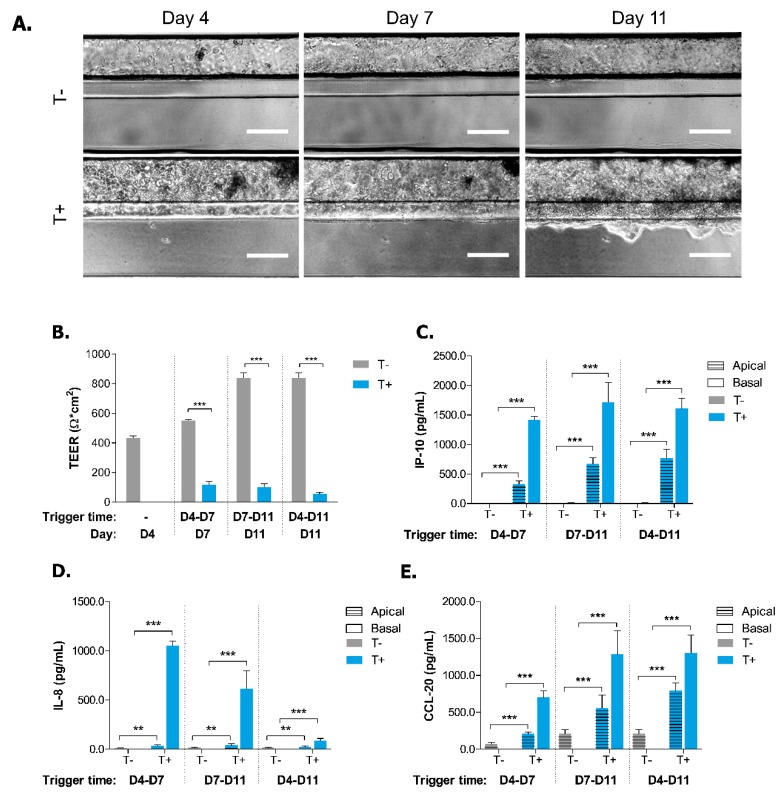
Effect of short and long cytokine trigger on morphology and integrity of Caco-2 tubules. (**A**) Representative 4X phase contrast images of triggered (T+) and non-triggered (T-) Caco-2 tubules at Days 4, 7 and 11. Scale bars = 100 µm. (**B**) TEER values of triggered (T+) and non-triggered (T-) Caco-2 tubules at Days 4, 7 and 11. Data is presented as mean ± SEM. ** *p* ≤ 0.01; *** *p* ≤ 0.001 by two-way ANOVA with Bonferroni corrected post-hoc test compared to T- of each time point (*n* = 3–10). (**C**–**E**) Secretion of IP-10 (**C**), IL-8 (**D**) and CCL-20 (**E**) in apical and basal compartments of triggered (T+) and non-triggered (T-) Caco-2 tubules at Days 7 or 11. Data is represented as mean ± SEM. * *p* < 0.05; ** *p* < 0.01; *** *p* < 0.001 by two-way ANOVA with ArcSinh transformation and Holm corrected post-hoc test and compared to apical and basal T- of each time point (*n* = 3–5).

We consequently adapted the concentration of the trigger to the OrganoPlate platform. Because of the difference between the Transwell and the OrganoPlate systems, which is mainly explained by the medium diffusion rate through the ECM gel in the OrganoPlate and in order to recapitulate the effects seen in the Transwell system, concentrations of 2, 100 and 100 ng/mL were set for IL-1β, TNF-α and IFN-γ, respectively (results not-shown). Caco-2 cells were triggered basally at Day 4 or Day 7 leading to short or long trigger times. Overall, the morphology of Caco-2 cells did not change upon trigger. However, Caco-2 cells submitted to a prolonged inflammatory trigger frequently started to invade the ECM (Figure 2A). As a change in the epithelium permeability is often an important event in IBD etiology [35], we assessed whether the cytokine trigger affected the TEER values of the tubules. The TEER values increased over time in non-triggered Caco-2 tubules (Figure 2B). However, upon triggering, the TEER values of the Caco-2 tubules decreased significantly for all trigger times when compared to non-triggered conditions. The prolonged trigger resulted in the lowest TEER values (Figure 2B). 

To assess the effect of the inflammatory trigger on the cellular activation of Caco-2 cells, the production of epithelial cytokines IP-10, IL-8 and CCL-20 were quantified. Caco-2 cells secreted low amounts of these epithelial cytokines in non-triggered conditions (Figure 2C–E). After trigger, both apical and basal secretion of all analyzed cytokines was increased significantly, with no major differences between short and long trigger times (Figure 2C–E). However, the effect of the long trigger on secretion of IL-8 was marginal (Figure 2D). In summary, both short and long inflammatory triggers induced a loss of barrier function of Caco-2 tubules as well as an increased cell activation, depicted with an elevated cytokine production in both apical and basal compartments. 

**Figure 3:**
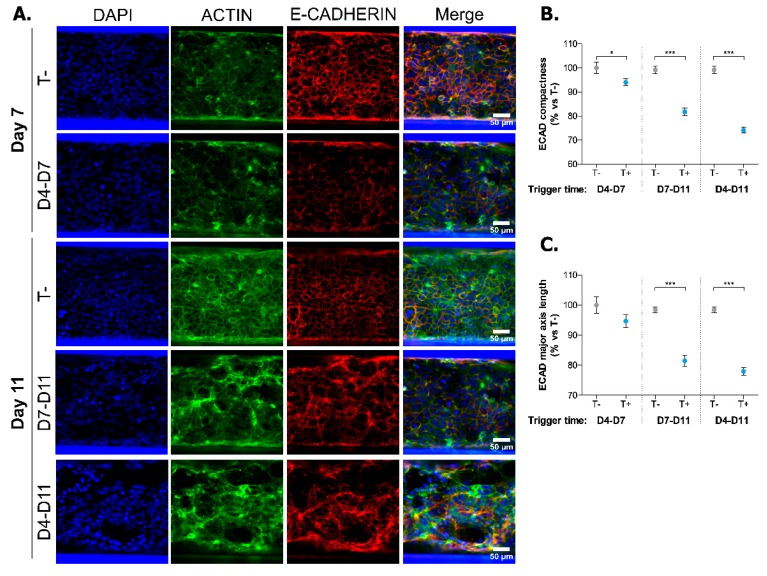
Short and long cytokine triggers induce morphological changes in Caco-2 tubules (**A**) Representative 20X images of Caco-2 tubules stained for cytoskeleton marker ACTIN, *zonula adherens* marker E-CADHERIN and nucleus marker DAPI at Day 7 and Day 11 in non-triggered (T-) or triggered (T+; D4–D7, D7–D11, D4–D11) conditions. Scale bars = 50 µm. (B,C) Compactness (**B**) and major axis length (**C**) of E-CADHERIN (ECAD) staining normalized to T- at Day 7. Data is represented as mean ± SEM. * *p* < 0.05; *** *p* < 0.001 by two-tailed Student’s *t*-test and compared to T- of each time point (*n* = 8–14). Segmentation process of ECAD staining is showed in Figure A3.

In an attempt to further understand the impaired TEER values of the Caco-2 cells upon trigger, we investigated the expression levels and localisation pattern of the *zonula adherens* protein E-CADHERIN (ECAD). It has been reported that in vitro wounded HT-29 monolayer models as well as CD and UC tissue have reduced levels of ECAD membranous expression [36,37,38]. To determine if this also occurs in our model, we stained Caco-2 cells for ECAD and the cytoskeleton marker ACTIN (Figure 3A). The organisational pattern of the ECAD staining was segmented and quantified based on two characteristics: compactness and major axis length of signal. A disorganized epithelial cell layer will display a fragmented ECAD phenotype with short major axes and low compactness values. Short and prolonged triggers both induced a significant reduction of these two characteristics in Caco-2 cells (Figure 3B,C). The compactness of the ECAD signal also showed a reduction following the early short trigger (D4-D7), but for this condition there was no significant effect on the length of the major axis. The reduction in epithelial cell layer organization confirmed the reduced TEER values of the triggered tubules. These results highlight that IBD-like conditions such as loss of barrier function and cytokine production can be induced in Caco-2 cells using a relevant cytokine trigger. 

### 2.3. Exposure to TPCA-1 Prevent the Inflammatory State of Caco-2 Tubules

In order to confirm the validity of our model for drug discovery purposes, we treated Caco-2 cells to a well-known anti-inflammatory compound, TPCA-1. TPCA-1 is a selective inhibitor of human IκB kinase-2 (IKK-2) [30]. Under normal conditions, IKK-2 phosphorylates the inhibitor of NF-κB (IκBα). When phosphorylated, IκBα releases NF-κB allowing its nuclear translocation to activate transcription of numerous genes involved in inflammation. Therefore, by inhibiting IKK-2, TPCA-1 prevents the nuclear translocation of NF-κB, leading to an anti-inflammatory effect. 

**Figure 4:**
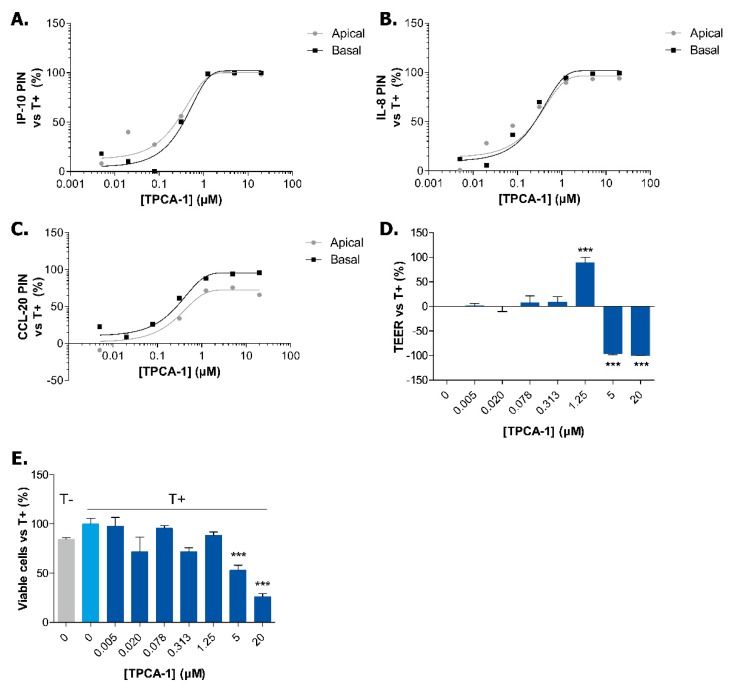
TPCA-1 exposure decreases cytokine secretion of Caco-2 tubules in a dose-dependent manner. (A–C) Percentage of inhibition (PIN) of IP-10 (**A**), IL-8 (**B**) and CCL-20 (**C**) secretion by Caco-2 cells at Day 7 following a 72 h TPCA-1 exposure in apical and basal compartments. Dots represent the PIN mean normalized to triggered (T+) but TPCA-1 untreated tubules. The line depicts a non-linear regression between [TPCA-1] and cytokine secretion (*n* = 4–5). (**D**) TEER values of TPCA-1 treated tubules at Day 7. Data is represented as percentage of triggered (T+) but TPCA-1 untreated tubules ± SEM. *** *p* < 0.001 by one-way ANOVA with Dunnett’s post-hoc test compared to triggered (T+) but TPCA-1 untreated tubules (*n* = 3–5). (**E**) Percentage of viable cells after TPCA-1 treatment at Day 7. Data is represented as percentage of triggered (T+) but TPCA-1 untreated tubules ± SEM. *** *p* < 0.001 by one-way ANOVA with Dunnett’s post-hoc test compared to triggered (T+) but TPCA-1 untreated tubules (*n* = 3–4).

Cells were exposed to TPCA-1 for two hours before a 72 h pro-inflammatory trigger was added in the continued presence of the compound. We determined the cytokine production levels in both the apical and basal supernatants. TPCA-1 induced a concentration-dependent inhibition of both apical and basal secretion of IP-10, IL-8 and CCL-20 by activated Caco-2 cells (Figure 4A–C). At concentrations of 5 and 20 µM, TPCA-1 could inhibit the secretion of all analytes to levels lower than non-triggered cells (results not shown). However, these higher TPCA-1 concentrations also suppressed the barrier integrity (Figure 4D) and the viability of the cells (Figure 4E). At a concentration of 1.25 µM, TPCA-1 led to a high percentage of inhibition (PIN) of cytokine production without significantly altering cell viability while restoring the barrier function of Caco-2 cells, when compared to triggered but TPCA-1 untreated tubules. These results clearly show that TPCA-1 exposure drastically decreases apical and basal cytokine secretion by Caco-2 cells and proves the suitability of the system to perform future compound exposure studies.

### 2.4. Adenoviral Knockdown of Inflammatory Effectors Prevents IBD-like Phenotype in Caco-2 Tubules

To further evaluate whether the inflamed state of Caco-2 cells could be prevented, we designed and tested several putative negative- and positive-control recombinant shRNA-expressing adenoviruses (AdV; Table A1 and Table A2) based on their ability to reduce CCL-20 production by Caco-2 cells (Figure A4). The results allowed us to select two non-targeting AdV (AdV-shmmNr1h3, AdV-shluc) and two AdV expressing validated shRNAs against MYD88 and RELA (AdV-shMYD88, AdV-shRELA) to be used in this study (Table 1). RELA is a subunit of NF-κB, a main player in inflammatory pathways that controls the expression of various pro-inflammatory genes such as chemokines, cytokines and adhesion molecules [39]. MYD88 is an adaptor protein downstream in the Toll-like receptor (TLR) and IL-1 signaling pathway involved in innate immune responses [39].

**Figure 5:**
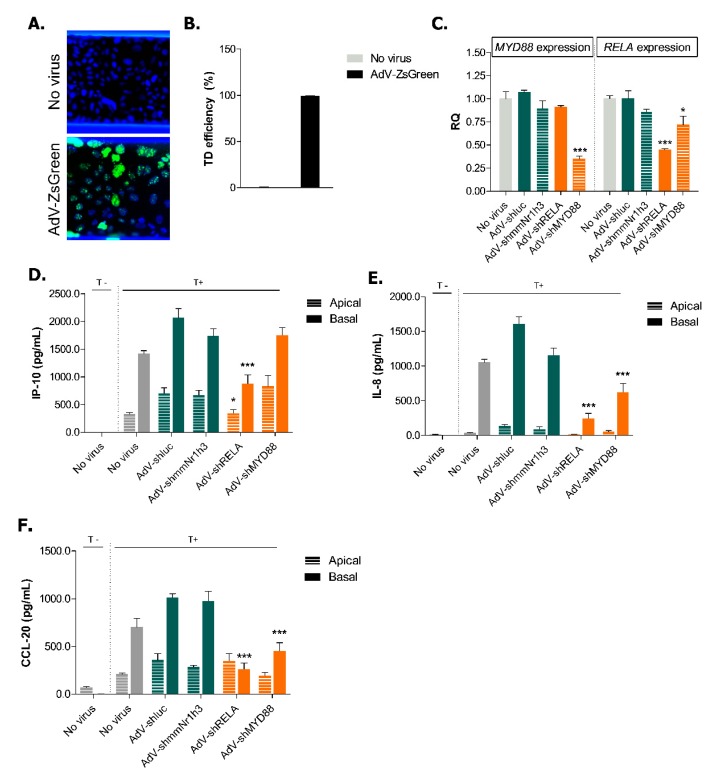
Efficient knockdown of inflammatory effectors decreases basal cytokine secretion in triggered Caco-2 tubules. (**A**) Representative 10× pictures of Caco-2 cells 72 h after on-chip transduction with a ZsGreen-carrying virus (AdV-ZsGreen). Green shows ZsGreen signal and blue shows DAPI signal. (**B**) On-chip transduction (TD) efficiency 72 h post-transduction. The number of ZsGreen-positive cells was quantified in 2 fields of 10X per chip and normalised to the number of total nuclei. Data is represented as mean ± SEM (*n* ≥ 4). (**C**) Knockdown efficiency of *RELA* and *MYD88* at Day 11. Data is represented as mean ± SEM normalised to *GAPDH* expression. * *p* < 0.05; *** *p* < 0.001 by two-way ANOVA with Bonferroni post-test compared to no virus condition (*n* = 2–3). (**D**–**F**) Secretion of IP-10 (**D**), IL-8 (**E**) and CCL-20 (**F**) in apical and basal compartments of non-triggered and triggered Caco-2 tubules at Day 7. Data is represented as mean ± SEM. * *p* < 0.05; *** *p* < 0.001 by two-way ANOVA with ArcSinh transformation and Holm corrected post-hoc test compared to AdV-shluc (*n* = 3–5). The non-transduced condition is shown in grey, negative control viruses in green and viruses carrying shRNA for inflammatory effectors in orange.

We chose to transduce cells directly in the chip. Recently, microfluidic transduction has been shown to be faster and more efficient than static transduction using clinically processed GFP-carrying lentiviruses [40]. We demonstrate, for the first time, the use of direct on-chip adenoviral transduction to conduct knockdown studies. In order to evaluate the transduction efficiency of on-chip AdV-mediated delivery, Caco-2 cells were transduced with a recombinant AdV expressing a ZsGreen DNA (AdV-ZsGreen). Microscopic evaluation confirmed that the delivery of 5 infectious units (IU) of ZsGreen-carrying virus per cell resulted in almost 100% transduction of Caco-2 cells at Day 4 (Figure 5A,B). We thus concluded that transduction experiments could be efficiently performed directly in the OrganoPlate.

The knockdown efficiency was further assessed on Day 11 and confirmed a 65% reduction of *MYD88* mRNA expression by AdV-shMYD88 and a 55% reduction of *RELA* mRNA expression by AdV-shRELA (Figure 5C). Previous studies have shown that *Myd88*-knockout in murine bone marrow-derived macrophages show decreased levels of NF-κB p65 subunit (RELA) activation following *Enterococcus faecalis* infection [41]. This suggests that MYD88 might be involved at some level in the regulation of RELA expression or activation, which could in turn explain the 28% reduction of *RELA* mRNA expression following MYD88 knockdown (Figure 5C). 

After having confirmed that the adenoviral technology induces effective knockdown, we investigated how the knockdown of *RELA* and *MYD88* could alleviate the IBD phenotype in Caco-2 cells. We could not determine whether the knockdown could prevent the loss of barrier integrity as the adenoviral delivery of non-targeting constructs affected the TEER values of the Caco-2 tubules (Figure A5). We started by investigating if the cytokine production of Caco-2 cells in non-triggered conditions was affected. The adenoviral transduction itself induced the apical production of IP-10, CCL-20 and IL-8 by Caco-2 cells (Figure A6 and Figure 5D–F). However, when compared to the negative control AdV-shluc, *MYD88* knockdown reduced both the apical and basal production of IL-8 as well as the basal production of CCL-20 in non-triggered conditions (Figure A6). The knockdown of *RELA* reduced the apical production of IP-10, both the apical and basal production of IL-8 but did not affect CCL-20 production. However, the production of these pro-inflammatory cytokines in Caco-2 cells is low in non-triggered conditions (note the difference in the range of the Y-axis in Figure A6 in comparison to the triggered samples in Figure 5D,F). However, we were particularly interested to know whether we could prevent the IBD phenotype establishment in our model and we therefore analyzed cytokine production in triggered Caco-2 cells. Compared to AdV-shluc, AdV-shRELA significantly inhibited the apical and basal secretion of IP-10 (Figure 5D) as well as the basal secretion of both IL-8 and CCL-20 (Figure 5E,F). The reduced expression of *MYD88* also caused a decrease in secretion of basal IL-8 and CCL-20 (Figure 5E,F). We did not observe a complete reduction of cytokine production after *RELA* and *MYD88* knockdown likely due to partial redundancy in underlying signaling pathways leading to cytokine production and due to incomplete knockdown. However, we show that our model could lead to potential target discovery, even by using a trigger composed of a mixture of different cytokines.

## 3. Discussion

When developing new disease models, users presently often have to choose between the level of throughput they want to achieve and the physiological relevance of their model. From a drug development perspective, achieving a high level of throughput is an important factor to consider as it significantly decreases the time and costs associated with screening activities. The Organ-on-a-Chip technology offers a valid alternative that promises increased throughput while achieving higher physiological relevance when compared to current traditional membrane inserts such as Transwells. When compared to membrane inserts, the OrganoPlate platform used in this study decreases media and reagent consumption by up to 10-fold depending on the assay, while offering an increased scalability. Furthermore, it also decreased the experimental time needed to obtain a differentiated epithelium from 21 days to only 4 to 5 days [26]. In comparison with silicon-based microfluidic chips, the OrganoPlate offers an easy-to-handle technology on a 384-well plate format that is tube- and pump-free and compatible with all lab instruments and high content imagers. Furthermore, Organ-on-a-Chip technology could be of great interest to pharmaceutical companies as one recent estimate anticipates an overall reduction of 10% to 26% in total research and development costs if organs-on-a-chip are implemented in a standard drug development process [42,43,44].

In this study, we show a robust and reliable 3D gut-on-a-chip model that can be used in drug discovery. More importantly, by applying specific downstream cues of immune activation on Caco-2 cells, we were able to recapitulate key physiological aspects of IBD pathology; the loss of barrier integrity and an increased cytokine production. 

For the first time, we could simultaneously monitor the barrier integrity of 40 membrane-free Caco-2 tubules in a real-time manner by measuring the TEER values of the tubules. In non-triggered conditions, Caco-2 tubules could reach up to 600–800 Ω·cm^2^ in TEER values after 11 days of culture. Upon inflammatory trigger, we show a significant reduction in TEER values, highlighting the impaired barrier function of the tubules. In the Transwell system, the TEER values of Caco-2 monolayers generally vary between 250–400 Ω·cm^2^, but TEER values as high as 1200 Ω·cm^2^ have also been reported [33,45,46]. We have also observed slight variations in the TEER values of non-triggered Caco-2 tubules over different experiments. Variations in TEER values have been reported before due to factors such as temperature, medium formulation, cell culture period and passage number of cells [33]. This high variability in the range of TEER values of Caco-2 cells makes it difficult to fully integrate our results in the light of recent literature and to understand their physiological relevance. Recent studies have also started to further investigate the problematic in order to understand the variation in TEER values between the Transwell system and microfluidic platforms [47]. Nevertheless, this novel method allowed us to constantly monitor the barrier integrity of Caco-2 tubules in order to further validate the Caco-2 gut-on-a-chip model previously described [26], before establishing IBD-like conditions. 

It was previously shown that protein expression of IL-1β [48] and IFN-γ [49] as well as mRNA expression of *IFN-γ* [50,51] are upregulated in the mucosa of patients with active IBD. Our data clearly demonstrates that an inflammatory state, reflected by an increased cell activation and a decreased barrier function, can be induced in the Caco-2 gut-on-a-chip model following trigger with immune-relevant cytokines IL-1β, IFN-γ and TNF-α. Since cell activation and loss of barrier function are key inevitable events of IBD pathogenesis [52], the increased cytokine release by IECS and the drop of TEER values upon the induction of IBD-like conditions support the relevance of our model to study disease-specific mechanisms of IBD. Interestingly, Caco-2 cells secreted low cytokine levels in basal conditions as expected [53]. Upon inflammatory trigger, both apical and basal production of epithelial inflammatory cytokines IL-8, IP-10 and CCL-20 were increased. However, the basal secretion of these cytokines was consistently higher when compared to their apical production. As both cytokine receptors and pro-inflammatory molecules are localised and secreted basally in polarized IECs in order to recruit immune cells to the site of inflammation [53,54], the increased basal cytokine production by Caco-2 cells upon trigger therefore reflects the in vivo physiology of the gastrointestinal tract in inflamed conditions.

Furthermore, we showed that the decreased barrier function of Caco-2 cells was associated with a fragmented localisation of *adherens junction* (AJ) protein ECAD upon inflammatory trigger. Mislocalisation of ECAD has been observed in CD patients and may be linked with the decreased intestinal barrier function of these patients [55]. Tight junctions (TJ) have also been shown to be affected in IBD patients, with notably elevated levels of CLAUDIN-2 protein in colonic biopsies of UC patients [56]. To this day, it is still unclear whether the loss of barrier function is a cause or a consequence of the establishment of inflammation in IBD patients. It would therefore be interesting to use this model to investigate how and when AJ and TJ are affected in IBD pathology. 

Throughout our research, we used different inflammatory trigger timelines to induce IBD characteristics. The initial rationale behind the idea was that using a short or a long trigger time would induce different degrees of phenotype severity; the shorter trigger leading to a milder phenotype and the longer trigger leading to a more severe one. However, we did not observe any differences in cytokine production by Caco-2 cells between the short and the long triggers nor did we observe any striking differences in TEER values or ECAD localization. These results suggest that the increased cytokine production and the loss of barrier function in Caco-2 happen shortly after trigger and that the time of exposure does not further modulate these characteristics. In the future, the concentration of the cytokines used in the trigger could potentially be optimized in order to illustrate different levels of severity of the IBD phenotype in this gut-on-a-chip model. Nevertheless, we did observe that Caco-2 cells started to invade the ECM after a prolonged exposure to cytokines. It is known that many cytokines are able to regulate proliferation and invasion of Caco-2 cells and a prolonged exposure to IFN-γ, TNF-α and IL-1β might affect those processes [57].

Interestingly, when assessing the effect of the trigger on cell activation, we observed that a prolonged cytokine trigger did not increase IL-8 production by Caco-2 cells compared to non-triggered conditions. This could be explained by the fact that the triggering medium was refreshed at Day 7, before cytokine production levels were assessed at Day 11. Indeed, it is known that IL-8 is rapidly produced after TNF-α triggering of Caco-2 cells and that the secretion is stopped approximately 12 h after trigger [58]. It is plausible that, by refreshing the medium at Day 7, levels of IL-8 were back to normal and the exhausted Caco-2 cells could not produce any additional IL-8.

Finally, we showed the functionality of this simple gut-on-a-chip model in IBD target and drug discovery by preventing the effect of the IBD-like trigger through compound and gene knockdown intervention. We used direct on-chip transduction to knockdown the expression of *MYD88* and *RELA*. We showed that the increased cytokine production induced in triggered Caco-2 cells could be partially suppressed by *MYD88* and *RELA* knockdowns, making the system applicable for large-scale knockdown screens. Once again, it was noticed that the basal production of the analytes was more affected by the knockdown than the apical production. As IECs generally recruit immune cells to the damaged epithelium by secreting pro-inflammatory cytokines in the subjacent intestinal mucosa [53,54], these results support the physiological relevance of our model to study IBD mechanisms. Nevertheless, we observed residual effect of the trigger cocktail on cytokine production after *RELA* and *MYD88* knockdowns. This can first be explained by the partial knockdown induced by the shRNA-carrying viruses. Furthermore, the cytokines we quantified as readouts of the inflammation are involved in several of the pathways activated by the trigger cocktail and the knockdown of a single effector of these pathways is not sufficient to completely prevent cytokine release. Nevertheless, we showed that this gut-on-a-chip is suitable for target discovery, even in a set-up using a mixture of different cytokines, reflecting the actual pathological situation in IBD. 

The effect of the knockdown of inflammatory genes on barrier integrity could not be assessed as AdV transduction affected the TEER values of the Caco-2 tubules. The interaction between AdV and its receptors might have, amongst others, affected TJ or cell–cell interaction which may have impaired attachment of the cells and therefore, decreased TEER values. As MYD88 and RELA are immune regulators and are not expected to play a significant role in the maintenance of barrier integrity, it would be interesting to determine whether the knockdown of genes involved in barrier function such as *HNF4A* or *ECM1* could rescue the effect of AdV transduction on TEER values of Caco-2 tubules. Furthermore, we could potentially overcome this limitation of our model by establishing stable knockdown Caco-2 cell lines before seeding them in the OrganoPlate. 

Finally, by using a compound treatment we could not only prevent the increased release of inflammatory cytokines but also the loss of barrier function in triggered Caco-2 tubules. Namely, Caco-2 tubules treated with 1.25 µM of TPCA-1 retained their barrier integrity without affecting cell viability. The lower TPCA-1 concentrations did not significantly decrease cytokine release and the higher concentration caused cell death, which affected the barrier function of the epithelium. These results suggest that this model could be used to assess the effect of compounds on both cell activation and barrier integrity.

Our system shows the potential for further improvements by replacing Caco-2 cells with intestinal organoids or gut epithelium derived from iPSCs, thus making it more physiologically relevant. By including patient-derived material, we would be able to test and predict individual responsiveness to medication and find the optimal treatment for a given IBD patient. Research groups have started to use such primary cells in order to develop gut-on-a-chip models [22,59], but they are of limited throughput and their application to disease modeling and drug screening has yet to be performed. Overall, these types of models could be of tremendous help in the rise of the personalized medicine field. In addition to the culture of human primary material, the membrane-free OrganoPlate platform is also ideally suited for co-cultures with immune cells, adding further complexity and relevance. This would allow the elicitation of epithelial-immune crosstalk mechanisms and could be of particular interest if microbial products are added to the model. There is consensus that gut microbiota is directly engaging with immune cells in the intestinal tissue and that this interaction is an important factor contributing to IBD pathogenesis, however the nature of this causal relationship has yet to be confirmed [60,61,62]. By using a high-throughput platform that allows different microbial taxa to interact directly with the intestinal epithelium, together with unprecedented imaging capabilities and the possibility to model low oxygen levels, we could gain a deeper understanding of the relation between microbiota and IBD.

In summary, this study establishes for the first time a robust and reliable gut-on-a-chip model allowing the recapitulation of key aspects of IBD pathogenesis in a high throughput manner. This work provides a foundation for upcoming large-scale 3D microfluidic modeling of IBD and screening of relevant therapeutic targets, allowing us to further study and understand gut inflammation.

## 4. Materials and Methods 

### 4.1. Ethics Statement

The research described here has been performed according to applicable Dutch national ethics regulations and was conducted within Galapagos BV (Leiden, The Netherlands). Scientists from Galapagos BV are qualified to perform research using human material and have appropriate facilities and equipment available to comply with applicable laws, regulations and internal rules related to handling and storage of the material. The human material was obtained from Sanquin (Amsterdam, The Netherlands). The supplier has confirmed to Galapagos BV that informed consent from the donors to use the material for research purposes was received. The cells were solely used for target and drug discovery and were not used for human experimentation or therapy. All material is and will remain anonymized.

### 4.2. Cells

Human colon adenocarcinoma cell line Caco-2 (ECACC 86010202) was cultured in Caco-2 medium composed of EMEM (ATCC, Manassas, VA, USA) supplemented with 10% FBS (Gibco, Waltham, MA, USA), 1% NEAA (Gibco, Waltham, MA, USA) and 1% penicillin/streptomycin (Gibco, Waltham, MA, USA). Cells were cultured at 37 °C in a humidified atmosphere with 5% CO_2_ up to 80% confluency and then either sub-cultured or used for the experiments. All experiments were performed on cells between passages 47 and 60.

### 4.3. OrganoPlate Seeding and Tubule Formation

Detailed extracellular matrix (ECM) loading and seeding procedures of Caco-2 cells in three-lane OrganoPlate were performed as previously reported [26]. In summary, 20,000 Caco-2 cells were seeded against a pH-buffered 4 mg/mL Collagen I gel (Cultrex, Gaithersburg, MD, USA) in a 3-lane 400 μm OrganoPlate (Mimetas, Leiden, The Netherlands) and allowed to attach against the gel for 4 h (37 °C, 5% CO_2_). After attachment, Caco-2 medium was added to medium inlets and outlets and perfusion was started by putting the plate on an interval rocker (Perfusion Rocker Mini, Mimetas, Leiden, The Netherlands) switching between a +7° and −7° inclination every 8 min (37 °C, 5% CO_2_) thus allowing bi-directional flow. Medium was refreshed every 3 days post-seeding.

### 4.4. Triggering of IBD-like Conditions

Caco-2 cells were triggered basally with an adapted cocktail of human recombinant cytokines (ImmunoTools, Friesoythe, Germany) composed of IL-1β, IFN-γ and TNF-α at respectively 2, 100 and 100 ng/mL dissolved in Caco-2 media. Three trigger times were used throughout experiments (Day 4 to 7, Day 7 to 11 or Day 4 to 11); the Day 4 to 7 trigger is shown unless stated otherwise.

### 4.5. Cytokine Secretion of Caco-2 Cells

Media was harvested separately form the top and bottom inlets and outlets, reflecting the apical and basal secretion of analytes by Caco-2 cells, and stored at −20 °C until further assessment. The concentrations of macrophage inflammatory protein-3 (CCL-20/MIP3A), IFN-Gamma-Inducible Protein 10 (IP-10/CXCL10), Interleukin-8 (IL-8/CXCL8), Interleukin-6 (IL-6), Interleukin-1-Beta (IL-1β) and Tumor Necrosis Factor-Alpha (TNF-α) were quantified using a human multiplex assay (Thermo Fischer, Waltham, MA, USA) on a Luminex FlexMap 3D (Merck Millipore, Burlington, MA, USA) according to manufacturer protocol. 

### 4.6. TEER Measurements

Transepithelial electrical resistance (TEER) was measured at different time points using an automated multichannel impedance spectrometer designed for use with the OrganoPlate (OrganoTEER, Mimetas, Leiden, The Netherlands). Before measurement, medium was added in the gel inlets and outlets and the OrganoPlate was returned to the incubator (37 °C, 5% CO_2_) to equilibrate for an hour. The electrode board of the OrganoTEER is matched to the OrganoPlate such that when an OrganoPlate is placed in the OrganoTEER, electrode pairs are dipped in the medium in all inlet and outlet wells connecting to the basal and apical side of all tubes. Point impedance measurements were performed by frequency sweep from 5 Hz to 1 MHz (75 points at precision 0) with TEER values going up to 1000 Ω·cm^2^. Data was analyzed using OrganoTEER software, which automatically extracts the TEER contribution (in Ohm) from the measured spectra and normalises it to Ohm·cm^2^ by multiplying by the tubule-ECM interface (estimated at 0.0056 cm^2^).

### 4.7. Immunohistochemistry

Caco-2 tubules were fixed with 3.7% formaldehyde (Sigma-Aldrich, St. Louis, MI, USA) in PBS for 10 min and prepared for immunohistochemistry as previously described [26]. The primary antibody mouse a-E-CADHERIN (Abcam AB1416, 1:100, Cambridge, UK) and the secondary antibody donkey a-mouse AlexaFluor647 (Molecular Probes A31571, 1:250; Eugene, OR, USA) were used. Actin was stained using ActinGreen™ 488 ReadyProbes™ Reagent (Thermo Fisher, R37110, Waltham, MA, USA) and nuclei was stained using NucBlue™ Fixed Cell ReadyProbes™ Reagent (Thermo Fisher, Waltham, MA, USA). Both reagents were added in the secondary antibody solution according to the manufacturer’s instructions. All steps were performed at room temperature on an interval rocker (Perfusion Rocker Mini, Mimetas) switching between a +7° and −7° inclination every 1 min. Cells were imaged on the InCell 6000 (GE Healthcare Life Sciences, Marlborough, MA, USA) and the Micro XLS-C HCI System (Molecular Devices, San Jose, CA, USA).

**Figure A3:**
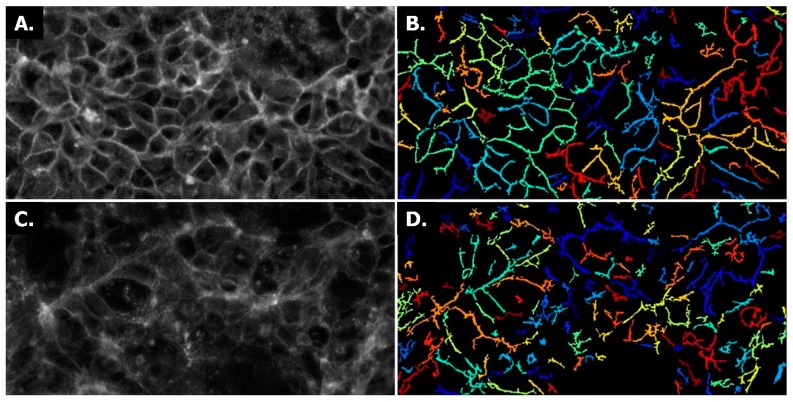
E-CADHERIN immunochemistry signal segmentation of triggered and non-triggered Caco-2 tubules using Cell Profiler. Raw input images of non-triggered (**A**) and triggered (**C**) Caco-2 tubules at Day 11 with their respective segmented masks (**B** and **D**) from the Cell Profiler analysis.

**Figure A4:**
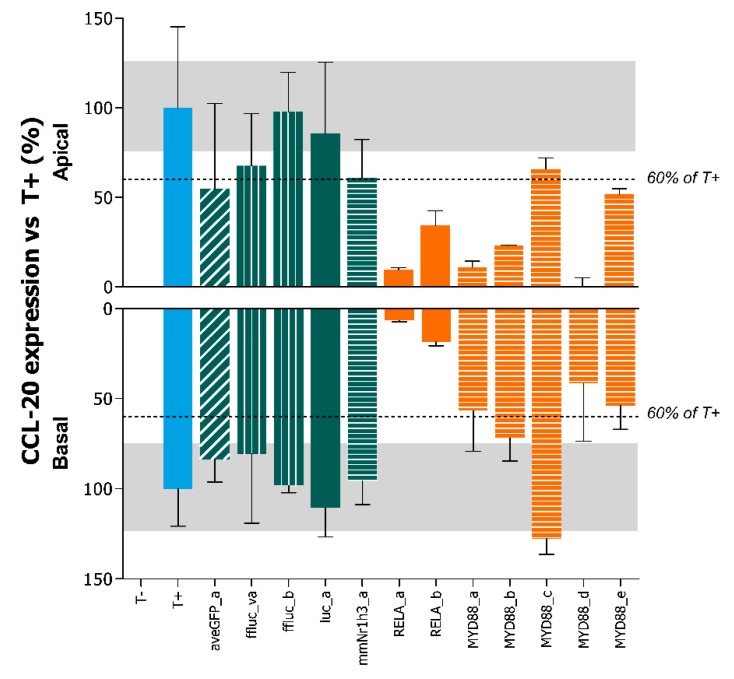
Selection of positive- and negative-control viruses based on CCL-20 production. Apical and basal production of CCL-20 by transduced Caco-2 cells (MOI 8–65) assessed by Luminex at Day 12 after a 72 h trigger with cytokines IL-1ß, TNF-α and IFN-γ (1, 50, 50 ng/mL respectively). Grey areas depict a 25% range from T+ and dotted lines depict a 40% reduction from T+. Data is represented as mean ± range of data (*n* = 2–4). Conditions with non-transduced cells are shown in blue, putative negative control viruses in green and putative positive control viruses in orange.

**Figure A5:**
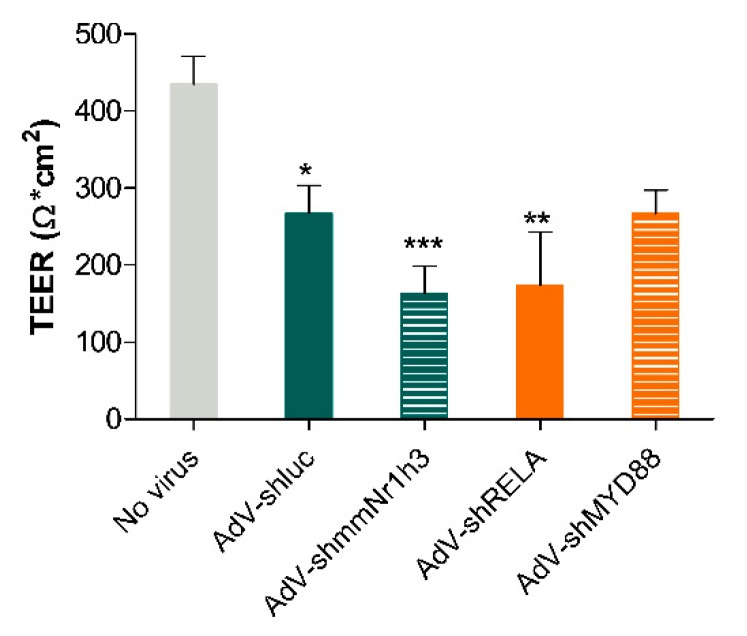
Adenoviral transduction of Caco-2 tubules cause a reduction of TEER values. TEER values of transduced Caco-2 tubules at Day 4. Data is presented as mean ± SEM. * *p* ≤ 0.05; ** *p* ≤ 0.01; *** *p* ≤ 0.001; **** *p* ≤ 0.0001 by one-way ANOVA with Bonferroni post-hoc test compared to No virus condition (*n* = 5–12).

**Figure A6:**
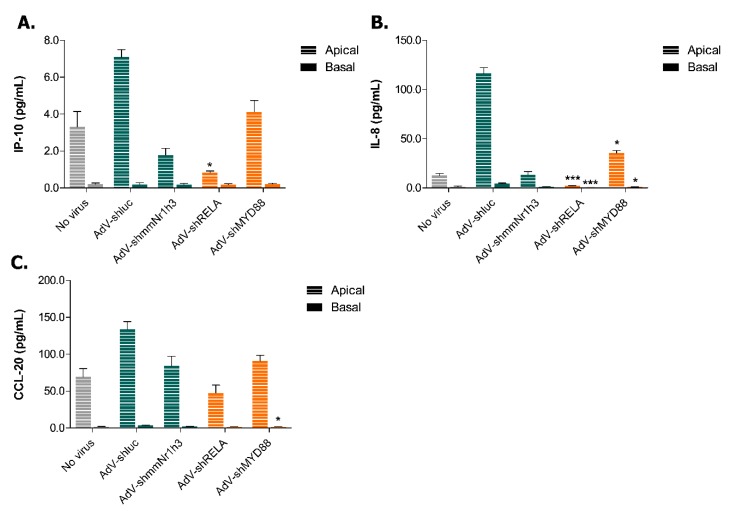
Knockdown of inflammatory effectors decreases basal cytokine secretion in non-triggered Caco-2 tubules. Secretion of IP-10 (**A**), IL-8 (**B**) and CCL-20 (**C**) cytokines in apical and basal compartments of non-triggered Caco-2 tubules at Day 7. Data is represented as mean ± SEM. *, *p* < 0.05; **, *p* < 0.01; ***, *p* < 0.001 by two-way ANOVA with ArcSinh transformation and Holm corrected post-hoc test compared to AdV-shluc (*n* = 3–5). Non-transduced condition is shown in grey, negative control viruses in green and viruses carrying shRNA for inflammatory effectors in orange.

The organization of the epithelial cell layer forming the Caco-2 tubule was quantified by segmentation of the E-CADHERIN (ECAD) positive cell junctions with Cell Profiler 3.0 [63]. ECAD signals were enhanced using a neurite tubeness method to amplify the junction patterns. Subsequently the junctions were identified with a three classes adaptive Otsu threshold. To reduce fragmentation, the objects were merged and filtered with area of >600 pixels. The MeasureObjectSizeShape module was used to extract non-intensity related features of the ECAD objects and the MeasureImageIntensity module for extraction of intensity-related measurements from the ECAD objects. Input images and final output segmentation overlays are printed in Figure A3. Based on this segmentation process, two characteristics of the organizational pattern of the ECAD staining were quantified: compactness and major axis length of signal. The compactness represents the mean squared distance of the object’s pixels from the centroid divided by the area. The major axis length represents the pixels’ length of the major axis of the ellipse that has the same normalised second central moments as the region. A more tightly packed and organized epithelial cell layer will have a more continuous pattern of tight junctions leading to objects with a long length axis and a high compactness value.

### 4.8. Adenoviral Preparation

For the generation of shRNA-carrying adenoviruses (AdV), pIPspAdapt-based constructs were transiently transfected with AdV5.Fib50 helper DNA into PER.C6/E2A producer cells as previously described [64,65]. The produced AdV were propagated by infecting PER.C6/E2A cells again. Throughout procedures, PER.C6/E2A cells were cultured in DMEM supplemented with 10% FBS (Gibco, Waltham, MA, USA) and 10 mM MgCl_2_. Finally, titers of the crude lysates were determined as described [66].

For transduction efficiency determination, a knock-in AdV for ZsGreen was used; The ZsGreen reporter-gene construct was adapted from the pZsGreen-C1 vector (Clontech, Kusatsu, Japan. A 3x NLS sequence was cloned directly downstream from the ZsGreen open reading frame for nuclear expression. The ZsGreen-3xNLS sequence was cloned into an AdV adapter plasmid containing a CMV promoter by standard restriction enzyme digestion and ligation.

Viruses were selected based on their ability to reduce CCL20 production by Caco-2 cells (see Appendix B).

### 4.9. Adenoviral Transduction

One day after seeding, Caco-2 cells were exposed apically to different shRNA-carrying recombinant AdV for 6 h (MOI of 5 IU/cell) in absence of penicillin/streptomycin on an interval rocker switching between a +7° and −7° inclination every 8 min (37 °C, 5% CO_2_). After transduction, medium was replaced with 50 µL of Caco-2 medium in medium inlets and outlets and plates were put back on the interval rocker (37 °C, 5% CO_2_) for the rest of the experiment.

### 4.10. Transduction Efficiency

Caco-2 cells treated with a ZsGreen-expressing virus were imaged 72 h after transduction to determine transduction efficiency. Cells were stained for 30 min at 37 °C with NucBlue™ Live ReadyProbes™ Reagent (Thermo Fisher, Waltham, MA, USA) following the manufacturer’s instructions and were then imaged on the InCell 6000 (GE Healthcare Life Sciences, Marlborough, MA). The transduction efficiency was calculated with an in-house algorithm quantifying the number of ZsGreen-positive cells relative to the number of total nuclei.

### 4.11. Quantitative PCR

Total RNA was isolated from Caco-2 tubules using a RNeasy Mini Kit (QIAGEN, Hilden, Germany) and converted to cDNA using TaqMan™ Reverse Transcription Reagents (Applied Biosystems, Waltham, MA, USA) according to the manufacturer’s instructions. Real-time quantitative amplification (qPCR) was then performed using TaqMan^®^ Fast Advanced MasterMix (Applied Biosystems, Foster City, CA, USA) on a LightCycler^®^ 480 instrument. Probes for MYD88 (Life Technologies, Hs01573837_g1; Carlsbad, CA, USA), RELA (Life Technologies, Hs00153294_m1) and GAPDH (Life Technologies, Hs02758991_g1; Carlsbad, CA, USA) were used.

### 4.12. Compound Exposure

Four days after seeding, Caco-2 cells were pre-treated apically and basally with TPCA-1 (Sigma-Aldrich, St. Louis, MI, USA) compound in a concentration range from 0.005 to 20 µM in a final DMSO concentration of 0.22%. After a pre-exposure of 2 h, basal medium was removed and replaced with cytokine-triggering medium (IL-1β, IFN-γ and TNF-α at 2, 100 and 100 ng/mL, respectively) in presence of the compound for another 72 h.

### 4.13. Viability Assay 

AlamarBlue Cell Viability Reagent (Thermo Fisher, Waltham, MA, USA) was pre-mixed in Caco-2 medium in a 1:10 ratio and 50 µL was added to medium inlets and outlets. Plates were incubated at 37 °C for 4 h and fluorescence signals were measured (Excitation: 530–560 nm, Emission: 590 nm) on a multi-well plate fluorimeter (Fluoroskan Ascent FL, Thermo Fisher, Waltham, MA, USA). 

### 4.14. Statistics and Data Analysis

Data was analyzed using GraphPad Prism software version 6 (GraphPad Software, La Jolla, CA, USA). Unless stated otherwise, values are expressed as mean ± standard error of the mean (SEM). When two groups of data were analyzed, a two-tailed, unpaired Student’s *t*-test was used to determine the statistical significance. When three or more groups were analyzed, parametrical or robust ANOVA were used to determine the statistical significance, depending on the presence of outliers. Differences with *p ≤* 0.05 were considered significant (*ns p* > 0.05, * *p* ≤ 0.05, ** *p* ≤ 0.01, *** *p* ≤ 0.001, **** *p* ≤ 0.0001). All graphs shown contain results from one representative experiment containing at least three technical replicates. The exact number of replicates in each condition is presented in the legend of each figure.

## Figures and Tables

**Table 1:** shRNA adenoviruses selected for the study.

Insert Name	Target Sequence
AdV-shluc	GGTTACCTAAGGGTGTGGC
AdV_shmmNr1h3	CACACATATGTGGAGGCCC
AdV_shMYD88	GGTTCATCACTGTCTGCGA
AdV_shRELA	GATTGAGGAGAAACGTAAA

## Abbreviations

AdV	Adenovirus
AJ	Adherens junction
CD	Crohn’s disease
DC	Dendritic cell
ECM	Extracellular matrix
IBD	Inflammatory bowel disease
IEC	Intestinal epithelial cells
iPSC	Induced pluripotent stem cell
NEAA	Non-essential amino acids
PBMC	Peripheral blood mononuclear cells
RQ	Relative quantification
TEER	Transepithelial electrical resistance
TJ	Tight junction
UC	Ulcerative colitis

## Appendix A. Supplementary Methods

### Appendix A.1. Cells

Peripheral blood mononuclear cells (PBMCs) were isolated from buffy coats (Sanquin, Amsterdam, The Netherlands) by standard Ficoll-Paque^TM^ density centrifugation and CD14^+^ cells were then positively-isolated using LS Columns from MACS Cell Separation system (Miltenyi, Bergisch Gladbach, Germany). Isolated CD14^+^ cells were cultured in RPMI supplemented with 10% heat-inactivated-FBS (Gibco, Waltham, MA, USA), 1% Penicillin/Streptomycin (Gibco, Waltham, MA, USA), [60 ng/mL] of GM-CSF (ImmunoTools, Friesoythe, Germany) and [40 ng/mL] of IL-4 (ImmunoTools, Friesoythe, Germany) for 7 days to differentiate towards dendritic cells (DCs).

### Appendix A.2. Optimization of the Immune-Relevant Trigger

An immune-relevant trigger was first set-up in the Transwell system (Corning, Figure A1 and Figure A2). 42,900 Caco-2 cells were seeded on top of a 96-well plate Transwell insert and were triggered at Day 16 with human recombinant cytokines IL-1β, IFN-γ and TNF-α (ImmunoTools, Friesoythe, Germany). Basal supernatants were harvested at Day 19 and cytokine secretion was assessed by Luminex. The trigger leading to the highest secretion of cytokines by Caco-2 cells (IL-1β, IFN-γ and TNF-α at 1, 50 and 50 ng/mL respectively) was then compared to the effect of UV-irradiated *E. coli*-activated DCs. To do so, 42,900 Caco-2 cells were seeded on top of a 96-well plate Transwell insert and DCs were added to the basal compartment in amounts ranging from 20,000 to 125,000 at Day 9. DCs were triggered with UV-irradiated *E. coli* (ATCC, #25922; Manassas, VA, USA) until Day 19 where apical and supernatants were harvested and cytokine secretion was assessed by Luminex.

### Appendix A.3. Selection of Viruses

Several putative positive- and negative-control AdV were screened for their ability to reduce CCL-20 secretion by Caco-2 cells seeded on top of a Transwell insert and triggered at Day 9 with a cytokine cocktail (IL-1β, IFN-γ and TNF-α at 1, 50 and 50 ng/mL respectively, Figure A4). Putative positive-control viruses needed to reduce CCL-20 secretion by at least 40% compared to non transduced conditions whereas CCL-20 expression of Caco-2 cells transduced with putative negative-control viruses could not differ from non-transduced conditions by more than 25%. Based on those criteria, luc_a and mmNr1h3_a were chosen as negative control AdV and MYD88_d and RELA_a as positive control AdV.

**Table A1:** Putative negative-control shRNA viruses tested.

Insert Name	Target Sequence
**aveGFP_a**	GCCACAACGTCTATATCAT
**ffluc_a**	GCAGAAGCTATGAAACGAT
**ffluc_b**	GCATGCCAGAGATCCTATT
**luc_a ***	GGTTACCTAAGGGTGTGGC
**mmNr1h3_a ***	CACACATATGTGGAGGCCC

* Virus selected for this study.

**Table A2:** Putative positive-control shRNA viruses tested.

Insert Name	Target Sequence
**MYD88_a**	GGAACAGACAAACTATCGA
**MYD88_b**	GGTTGTCTCTGATGATTAC
**MYD88_c**	GACTTCCAGACCAAATTTG
**MYD88_d ***	GGTTCATCACTGTCTGCGA
**MYD88_e**	CTGCTCTCAACATGCGAGT
**RELA_a ***	GATTGAGGAGAAACGTAAA
**RELA_b**	GTACCCTGAGGCTATAACT

* Virus selected for this study.
